# Comparative Analysis of Qualitative and Bioactive Compounds of Whole and Refined Flours in Durum Wheat Grains with Different Year of Release and Yield Potential

**DOI:** 10.3390/plants12061350

**Published:** 2023-03-17

**Authors:** Valeria Menga, Valentina Giovanniello, Michele Savino, Antonio Gallo, Salvatore Antonio Colecchia, Vanessa De Simone, Silvia Zingale, Donatella Bianca Maria Ficco

**Affiliations:** 1 Consiglio per la Ricerca in Agricoltura e l’Analisi dell’Economia Agraria—Centro di Ricerca Cerealicoltura e Colture Industriali, S.S. 673 km 25.200, 71122 Foggia, Italy; 2 Department of Agriculture, Food and Environment (Di3A), University of Catania, Via S. Sofia 100, 95123 Catania, Italy; silvia.zingale@phd.unict.it

**Keywords:** durum wheat, wholemeal flour, semolina, yield-related traits, phenolic compounds, phenolic acids, quality, pasting properties

## Abstract

Durum wheat varieties are important sources of nutrients and provide remarkable amounts of phytochemicals. Especially, phenolics, which are mostly located in external layers of grains, have recently gained increased interest due to their high antioxidant power. This study aimed to evaluate the differences in the quality traits and phenolic compounds’ concentration (e.g., phenolic acids) of different durum wheat genotypes, namely four Italian durum wheat cultivars and a USA elite variety, in relation to their yield potential and year of release. Phenolic acids were extracted both from wholemeal flour and semolina and analysed through HPLC-DAD analysis. Ferulic acid was the most represented phenolic acid, both in the wholemeal flour (438.3 µg g^−1^ dry matter) and in semolina (57.6 µg g^−1^ dry matter) across all cultivars, followed by *p*-coumaric acid, sinapic acid, vanillin, vanillic acid, syringic acid, and *p*-hydroxybenzoic acid. Among the cultivars, Cappelli showed the highest phenolic acid content, whilst Kronos had the lowest one. Negative correlations occurred between some phenolic acids and morphological and yield-related traits, especially for Nadif and Sfinge varieties. On the contrary, durum wheat genotypes with low yield potential such as Cappelli accumulated higher concentrations of phenolic acids under the same growing conditions, thereby significantly contributing to the health-promoting purposes.

## 1. Introduction

Although durum wheat accounts for only 5% of global wheat production, it represents the 10th most important cultivated cereal worldwide, especially in the Mediterranean region, and it is used to produce many high-quality traditional foods [1]. The latter are diversified based on the producing country: for instance, pasta is the symbol of the Italian culture, as well as bread, which is typically produced in Southern Italy, and also in Spain, and Turkey; whilst couscous, bulgur, and freekeh are particularly important in North Africa and the Middle-East, respectively.

However, durum wheat is primarily used for pasta-making purposes, and its production, processing, and trade conditions are regulated by specific standards, such as the Italian legislation (Italian Law 9 February 2001, No. 187), which specifically states that pasta must be made with only durum wheat semolina or durum wheat wholemeal semolina pasta, and water.

Furthermore, the requirement for high-quality pasta includes high carotenoid pigment content (mainly lutein), which provided the yellowness that is expected from consumers for pasta, and high protein content and gluten strength, which are associated with pasta firmness, and limited cooking loss values [2].

Moreover, besides gluten proteins, starch also plays an important role in pasta cooking quality, as its structure and composition highly influence the starch gelatinization process and protein network formation. In this regard, measuring starch gelatinization and pasting properties through micro visco-amilograph tests is an easy way to assess the final product quality [3].

In addition, durum wheat grains also contain polyphenols, including flavonoids and phenolic acids, which are important biomolecules contributing to plant responses to abiotic and biotic stresses [4], end-product quality such as colour, aroma, and taste and potential beneficial implications in human health, due to their antioxidant activity [5,6].

In particular, phenolic acids represent the most abundant class of polyphenols present in the cortical layer of grains, with ferulic acid accounting for about 90% of total phenolic acids, thus being the most representative one [7]. Phenolic acids may exist in free, soluble conjugated, and insoluble-bound forms. In particular, conjugated and bound phenolic acid may also play an essential role in delivering antioxidants to the colon upon their release by bacterial microbiota [5]. In mature durum kernels, phenolic acids are mainly present in the insoluble bound form, linked to cell wall structural components such as cellulose, lignin, and proteins through ester bonds [8]. The content and composition of phenolic acids have revealed significant differences among species, varieties, and grain fractions [8]. Despite this, durum wheat’s nutritional and health properties have been poorly considered in both breeding programs and food development processes, as much more attention has been always given to high yield and improved technological quality objectives.

Similarly, whilst strict quality standards are required by the Italian pasta sector in terms of ash and protein contents, gluten strength and yellow pigments’ concentration, to assure optimal rheological and technological performances, the same does not occur in the case of nutritional and health-promoting properties.

This calls for holistic and integrated studies to identify the possible trade-offs between productivity, technological quality, and nutritional and/or health aspects. Indeed, multiple factors influence the quality standards for Italian pasta, including the introduction of foreign cultivars with higher potential yield and quality (such as Kronos in this paper, e.g., a semi-dwarf durum wheat cultivar developed by Arizona plant breeders [9]) used in blend for pasta-making, and changes in processing techniques could determine both enhancements or detriments of the technological, nutritional and sensory properties of the final product.

Within this context, this study aims to:

- characterize different durum wheat genotypes, including four Italian varieties selected in the South of Italy, and an elite USA variety, in terms of quality traits and bioactive compounds, considering both wholemeal and semolina products;
- provide novel insights about the correlation between such features and their relation with the genotypes’ yield potential and year of release.

With specific regard to polyphenols, widely investigated within the specialized literature for their role in protecting plants against abiotic stresses, such as drought or increased temperature [4], this is the first time that a study has evaluated their relationship with quality and grain yield-related traits. In addition, the study provides useful insights into durum wheat pasting properties, as important quality traits from a processing perspective.

## 2. Results and Discussion

### 2.1. Whole Seed Morphological and Yield-Related Traits in Durum Wheat Genotypes

The genotypes were significantly different for all traits (*p* < 0.05) (Table 1). Nadif showed the highest kernel length, width and thickness associated with greater TW and TKW, whilst the opposite was observed in Marco Aurelio. The elite variety, Kronos, most cultivated in South Africa [9], was similar to Cappelli in thickness and the yield-related traits. The data were in the range previously observed for old and modern varieties [10,11].

### 2.2. Quality Traits of Wholemeal and Semolina Samples

Regarding the qualitative parameters of wholemeal, the protein content (PC) varied in the range comprised between 124 g kg^−1^ and 183 g kg^−1^ (Table 2). Cappelli and Marco Aurelio performed the highest values of PC, followed by Kronos, whilst the lowest values were recorded for Nadif and Sfinge.

In general, a negative association between protein content and yield-related traits was found and considered as a consequence of the improvement in grain yield of modern durum wheat varieties and the subsequent increase in harvest index and grain number per unit of surface, as discussed by Giunta et al. [12]. According to Lovegrove et al. [13], such high yield potential of modern cultivars is also associated with an increased accumulation of starch that possibly dilutes other components.

However, similarly to Carucci et al. [14], although different from Cappelli, the modern genotype Marco Aurelio showed a high protein content and adaptability to different conditions, besides high values of SDS and gluten index (see Table 2 and Table 3).

In line with Fares et al. [15], the SDS-sedimentation volume (SDS) ranged from 23.9 mL g^−1^ to 40.3 mL g^−1^. Therefore, contrary to what was observed for PC, the SDS, which is a widely used test for indirectly measuring the gluten strength of flours, was highest in Marco Aurelio and Kronos and lowest in Cappelli.

In addition, in semolina, the genotypic variations in the quality and bioactive compounds are listed in Table 3. The portion of total proteins represented by gluten showed a high variability among semolina samples. Gluten content (GC) follows the same trend as protein content, due to its strong correlation with PC (R = 0.97, *p* < 0.05), with higher values in Cappelli, Marco Aurelio and Kronos rather than in Sfinge and Nadif.

By contrast, gluten strength—a major quality indicator being used as a specification in durum wheat and semolina trading—measured by gluten index (GI) showed the highest values in Marco Aurelio and Kronos and the lowest one in the genotype Cappelli, in line with that reported by Pasqualone et al. [16] for classifying flours based on their GI (weak for GI < 30, normal for GI in the range of 30–80 and strong for GI > 80). This confirmed that protein quantity and quality were affected by breeding in opposite directions, as the decrease in protein content in modern cultivars compared to old ones was counterbalanced by a corresponding increase in gluten strength [17]. Moreover, our study also confirmed the positive correlation between GI and SDS (R = 0.99, *p* < 0.05), in accordance with the literature [16].

Considering the total carotenoids, a wide range was observed (3.92 µg g^−1^ to 9.32 µg g^−1^) with Marco Aurelio and Cappelli presenting the maximum and the minimum values. This confirms the strong selective pressure that has been exerted by breeders to increase the content of the carotenoid pigments in the modern durum wheat varieties [18]. Among colour indices, the yellow index (YI) was the predominant parameter due to the presence of carotenoid pigments that are strongly correlated with yellow hue [18]. Specifically, our data showed that the highest value of YI was in Marco Aurelio, followed by Kronos and Nadif. Instead, red index (RI) values were generally quite low, ranging from −0.95 to −3.16, with the highest values for Cappelli, also affecting the quality colour of end products.

As previously mentioned, durum kernels are also a source of health components, such as dietary fibre. Insoluble dietary fibre (IDF) is primarily found in the bran, whereas the soluble dietary fibre (SDF) fraction is mostly in the endosperm cell walls [19]. The highest values of IDF were recorded for Sfinge, whilst the lowest ones were in Marco Aurelio. Regarding the SDF, instead, the highest levels were reported for Cappelli and the lowest for Nadif. The highest SDF observed in Cappelli makes its consumption potentially useful for lowering the cholesterol level in the blood, by binding it in the small intestine, as stated by Ianiro et al. [20]. Moreover, the IDF and SDF data reported in this study are also in line with the range found by Khan et al. [21].

Some authors [20,22] reported that old varieties of wheat/durum wheat, in particular Cappelli, are characterized by great rusticity and adaptability to marginal soils along with a richness in secondary metabolites, including free and bound polyphenol isomers. Additionally, the properties of Cappelli would appear to be less influenced by environmental conditions than other varieties and this may impact gliadin content and consequent immunogenic potential [1]. Ianiro et al. [20] found, by a double-blind randomized cross-over trial, that consuming monovarietal pasta of Cappelli was effective in reducing symptoms in patients with non-celiac gluten sensitivity compared to standard pasta derived from a blend of varieties.

To compare the pasting properties of semolina samples, we used a micro visco-analyser for measuring viscosity as a function of time and temperature (Table 3).

The ANOVA indicated significant differences in the peak viscosity among samples (*p* < 0.05). Marco Aurelio and Nadif varieties showed the highest peak viscosity (1054 BU and 1018 BU, respectively). The peak viscosity is related to the degree of swelling of granules during heating, and is mainly affected by amylose content, and amylose: amylopectin ratio [23]. Such high starch peak viscosity values indicated that these two varieties’ starch granules tend to swell more, thus consequently reaching the desired texture, as observed in Japanese noodles by Crosbie et al. [24].

In addition, breakdown is a measure to describe starch granules’ ability to resist high temperature and shear stress. The decreased breakdown values found for Marco Aurelio and Nadif varieties (5 BU and 6 BU, respectively) confirmed their ability to support a higher resistance of starch granules to shear stress and disintegration at high temperatures [25]. Indeed, a lower breakdown value can be associated with a decrease in the percentage of broken starch granules, and consequentially to a greater stability after swelling. This could have a positive effect on the subsequent processing of semolina in pasta.

Regarding the setback viscosity, this parameter depends on the magnitude of starch retrogradation, e.g., the re-association between starch molecules during cooling. In this study, setback viscosity was high in all samples, except for Marco Aurelio (370 BU). This could indicate a lower tendency of Marco Aurelio semolina towards starch retrogradation than the other, thus assuming a potentially longer shelf-life for the respective derived products.

### 2.3. Variability of Antioxidants and Phenolic Acids in Wholemeal and Semolina Samples

**Figure 1:**
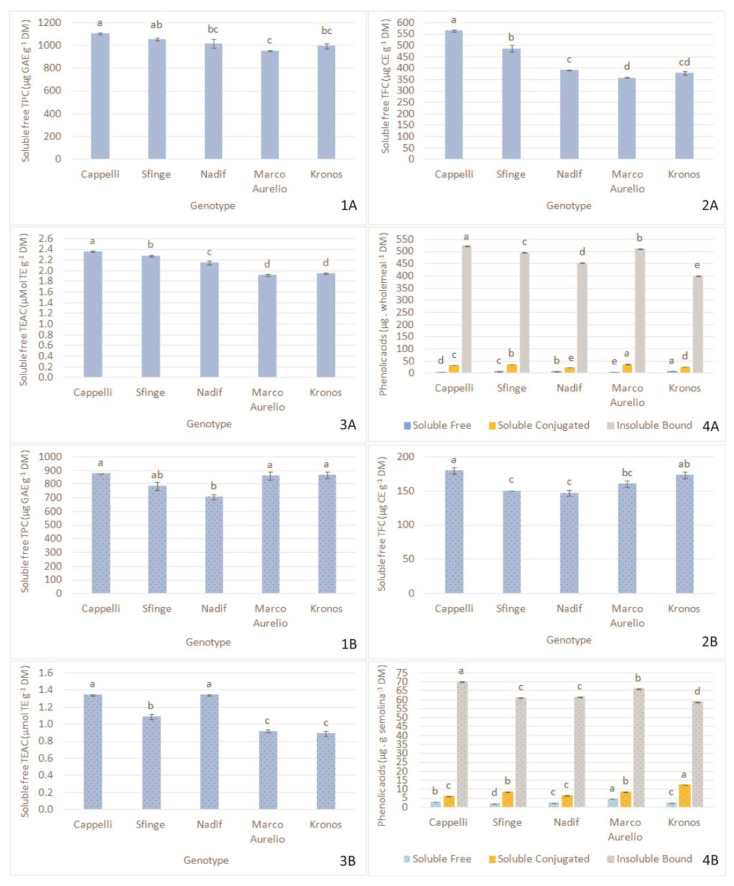
Soluble free total phenolic content (**1**), soluble free total flavonoid content (**2**), soluble free total antioxidant activity (**3**), sum of soluble (free and conjugated) and insoluble bound phenolic acids (**4**) of wholemeal (**A**) and (**B**) semolina of five durum wheat varieties. The same letter indicates no statistical difference, whereas different letters stand for significant statistical difference (*p* < 0.05; Tukey’s test). Legend: TPC, total polyphenol content; TFC, total flavonoid content; TEAC, total antioxidant activity.

Total polyphenol content (TPC) and total flavonoid content (TFC) are good contributors to the antioxidant activity of grains. TPC, TFC and total antioxidant activity (TEAC) of the five studied wholemeal samples are reported in Figure 1(1A–3A). The old variety Cappelli showed the highest levels of TPC and TFC and, consequently TEAC, whilst Marco Aurelio and Kronos were characterized by the lowest value of antioxidants.

Therefore, Cappelli was confirmed to be the genotype with the highest TPC, TFC and TEAC Figure 1(1B–3B), as reported also by Giacosa et al. [26], who assessed multiple nutraceutical functions for this genotype and suggested its use for health-promoting purposes. Interesting values of TPC were also evidenced for Marco Aurelio and Kronos, of TFC for Kronos and Marco Aurelio and of TEAC for Nadif.

Phenolic acids are the most common types of phenolic compounds in durum wheat. Figure 1 reported the sum of soluble and insoluble phenolic acids found in wholemeal (4A) and semolina (4B). The insoluble bound fraction prevails with significant variability both in wholemeal and semolina, in line with Vitaglione et al. [27], who observed differences due to variety, environmental conditions and milling process. As also reported by Andersson et al. [6], among the phenolic acids that occurred in free, conjugated or insoluble bound forms, about 75–80% of phenolic compounds are insolubly bound to cell wall polymers, 20–25% are esterified to sugars and other low molecular mass compounds, and only 0.5–2% are soluble and free. Fernandez-Orozco et al. [28] also found that environmental factors were more important determinants than genotypic variations of phenolics. This phenomenon was observed particularly in free and conjugated phenolic acid fractions and less in the insoluble form bound to dietary fibre (cell wall).

In general, phenolics in whole grain are unevenly distributed along the kernel but a gradient from the outside of the seed to the starchy endosperm was observed [29]. Therefore, the antioxidant potential is considerably higher in wholemeal. In fact, in our study, total phenolic acids were about seven-fold higher in wholemeal than in semolina in line with literature data [29]. Cappelli and Marco Aurelio reported the highest value of total phenolic acids both in wholemeal and semolina; the contrary was observed for Kronos. Additionally, Dinelli et al. [30] found significantly higher content of both free and bound polyphenol compounds in the old variety Cappelli in comparison to modern varieties.

Considering the composition of the insoluble bound form (Tables S1c and S2c), the main compound was represented by ferulic acid which accounts for up to 90% of total phenolic compounds in cereal grain with a content in our wholemeal samples (the mean 438.3 μg g^−1^) significantly higher than that in semolina (the mean 57.6 μg g^−1^), as wholemeal contains both bran layers and germ. Hung et al. [7] reported values of bound ferulic acid in the range of 368 to 605 μg g^−1^ for two Canadian wheat classes (whole wheat) of the sample. A variability among the genotypes was also observed for the other phenolic acids found that resulted in the range of literature [8].

In durum wheat grain flavonoids are not highly represented. Indeed, these metabolites, despite being important bioactive compounds with considerable antioxidant potential, are mainly found in coloured fruits, vegetables and grains, in which they effectively exert excellent antioxidant activity. In the study’s samples, a significant difference was observed only in vitexin content as free soluble form (Tables S1a and S2a).

Major levels of vitexin were observed in wholemeal according to the following order Marco Aurelio > Sfinge > Nadif > Kronos > Cappelli, whilst in semolina it showed descending levels in Cappelli > Kronos > Marco Aurelio. The data were in the range observed by other authors [31]. Vitexin is a C-glycosyl flavone with well-known high benefits to human health. It has been shown to inhibit alpha-glucosidase, an enzyme responsible for the breakdown of carbohydrates into sugar [32].

### 2.4. Correlation and Multivariate Analysis for Yield-Related Components, Quality and Phenolics

**Figure 2:**
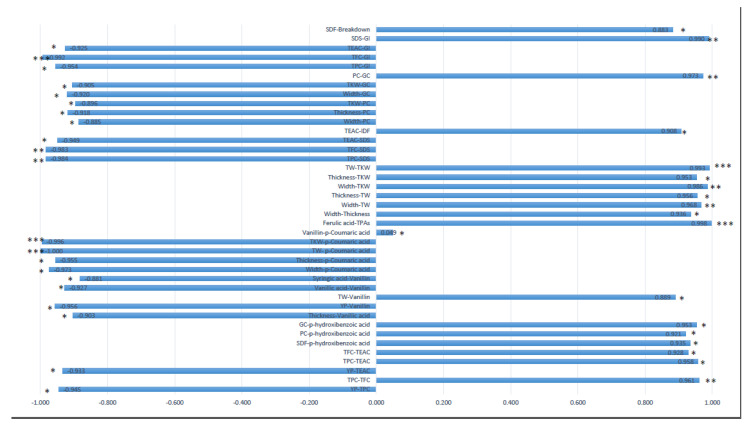
Pearson correlation coefficients among morphological and yield-related traits, quality, and bioactive compounds. Abbreviations: SDF, soluble dietary fibre; SDS, SDS-sedimentation volume; TEAC, total antioxidant activity; GI, gluten index; TFC, total flavonoid content; TPC, total polyphenol content; PC, protein content; GC, gluten content; TKW, thousand kernel weight; IDF, insoluble dietary fibre; TW, test weight; TPAs, total phenolic acids; YP, total carotenoid content. Significance level: ***, 0.001; **, 0.01; *, 0.05.

The Pearson correlation coefficients were calculated among morphological and yield-related traits, quality, TPC, TFC, YP, individual phenolic acids and total phenolic acids (TPAs) (Figure 2).

Two individual phenolic acids showed a correlation with grain yield-related components. In particular, *p*-coumaric acid was negatively correlated with TKW and TW (***) and with the thickness (*) and width (*). Similarly, vanillic acid was negatively correlated with the thickness (*). Based on the correlation with quality traits, hydroxybenzoic acid was positively correlated with GC, PC, and SDF (*). Additionally, breakdown was positively correlated with SDF (*). Moreover, considering the correlations among individual phenolic acids, vanillin was positively correlated with *p*-coumaric acid whilst ferulic acid showed a positive correlation with TPAs.

**Figure 3:**
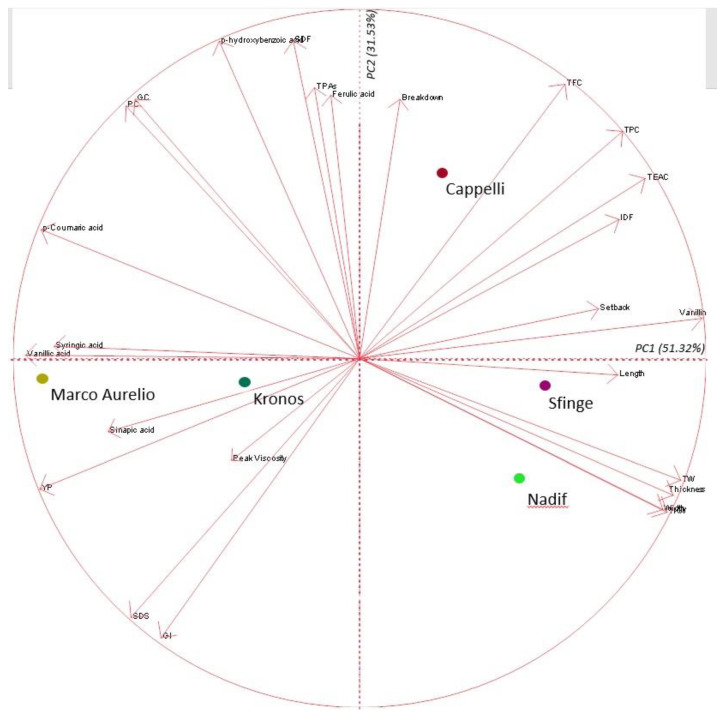
Biplot of the first (PC1) and second (PC2) principal components showing the variation for 26 traits. Genotypes are represented by different coloured symbols. Trait contributions are shown with arrows. The direction and distance from the centre of the biplot indicate how each trait contributes to the first two components. Abbreviations: TFC, total flavonoid content; TPC, total polyphenol content; TEAC, total antioxidant activity; IDF, insoluble dietary fibre; TKW, thousand kernel weight; TW, test weight; SDS, SDS-sedimentation volume; GI, gluten index; YP, total carotenoid content; TPAs, total phenolic acids.

To specifically evaluate the associations between the varieties and the investigated variables, a PCA was performed, and the responses were translated into a biplot (Figure 3).

The principal component 1 (PC1) explained 51.32% of the total variance, whilst the principal component 2 (PC2) was able to explain 31.53% of the variance, totalizing 82.85%. Breakdown, TFC, TPC, TEAC, IDF, setback, length, TW, thickness, width and TKW were mainly influenced by PC1, whereas PC2 was mostly attributed to GI, SDS, viscosity, YP, sinapic acid, vanillic acid, syringic acid, *p*-coumaric acid, PC, GC, hydroxybenzoic acid, TPAs and ferulic acid. PC1 also discriminated four of the five durum wheat genotypes.

In the biplot, three groupings have been identified:

- the first one, with some phenolic acids such as YP, sinapic acid and SDS being part of the same cluster, and the related genotypes Marco Aurelio and Kronos.
- The second one, with the morphological and yield-related traits such as length, thickness, width, TW and TKW being associated with Sfinge and Nadif.
- The third one, with antioxidants such as TFC, TPC, TEAC and the breakdown quality trait related to Cappelli.

Concerning this, it was interesting to identify in the correlation and multivariate analysis that when some phenolic acids increase, the grain width, the grain thickness, test weight and thousand kernel weight decrease, indicating that yield-related traits are at odds with high phenolic acid concentration. This result was more evident for the modern genotypes and, among them, for Nadif and Sfinge, which resulted to be more related to morphology and yield-related components [14]. Instead, durum wheat genotypes with low yield potential as Cappelli could accumulate greater amounts of phenolic acids under the same growing conditions, as also described by Serban et al. [33] for ancient wheats as more “healthy” than modern wheats in relation to their nutraceutical composition.

## 3. Materials and Methods

### 3.1. Plant Materials

Four Italian durum wheat (*Triticum turgidum* ssp. *durum* (Desf.), 2 n = 4× = 28; AABB genome) varieties, consisting of genotypes (Cappelli, Marco Aurelio, Nadif and Sfinge) originated in Southern Italy (Apulia) were chosen based on the year of release from 1915 to 2016 and subdivided into old and modern, the pedigree and the yield potential as reported in Table 4. The study was carried out on the farm of CREA-Centro di Ricerca Cerealicoltura e Colture Industriali in Foggia, Italy (41°28′ N, 15°32′ E; 75 m a.s.l.), over the growing season of 2020/2021, using a randomized complete block design, with three replicates. The field was managed with fertilizer applications following the local agronomic practices. Seeds were harvested at maturity and stored at 4 °C. In addition, the variety Kronos, a durum wheat cultivar with high yield potential and excellent pasta quality, selected in the USA and supplied by a local mill has been added for varietal comparison.

### 3.2. Wholegrain Analysis

Grain morphology (i.e., length, width, thickness (mm)) and thousand kernel weight (TKW; g) were determined using an Imaging System based on reflectance measurements (SeedCount SC5000R, Next Instruments, Condell Park, Australia). Test weight (TW) was determined for each sample by weighing a known volume of grain without hulls and impurities (250 g; shopper chondrometer) and expressed as kilograms per hectolitre (kg hL^−1^).

### 3.3. Milling

To assess the quality and phytochemical compounds in flours, kernels were ground to wholemeal using a laboratory machine (Tecator Cyclotec 1093, International PBI, Milan, Italy) with 0.5 mm hole sieve and to semolina by a Labormill 4 RB (Bona, Monza, Italy), after tempering the grain to 16.5% moisture.

### 3.4. Quality Traits Assessment

The protein content (PC) (*N* × 5.70) was assayed using the Dumas combustion nitrogen method, by FP528 (Leco Corp., Saint Joseph, MO, USA) and data were expressed as grams per kilogram dry matter (g kg^−1^ DM). The SDS-sedimentation volume (SDS) was expressed as millilitres per gram (mL g^−1^). Gluten content (GC) and gluten index (GI) were determined using the Glutomatic 2020 system (Perten, Sweden) and GC was expressed as grams per kilogram of dry matter (g kg^−1^ DM).

Insoluble dietary fibre (IDF) and soluble dietary fibre (SDF) were determined using a commercial kit (Megazyme International, Bray, Ireland) based on the enzymatic gravimetric procedure and expressed as grams per 100 g of dry matter (g 100^−1^ DM). Total carotenoid content (YP) was expressed as microgram per gram of dry matter (µg g^−1^ DM). All used methods are previously fully described by [15].

Colourimetric evaluations of yellow index (YI, corresponding to b*), red index (RI, a*), and brown index (BI, defined as 100-L*) were carried out by means of the reflectance colorimeter Chroma Meter (Konica Minolta Pty Ltd., Macquarie Park, NSW, Australia).

Pasting properties of semolina samples were measured using a micro visco-amylograph (Brabender OHG, Duisburg, Germany) according to Aalami et al. [34]. Fifteen grams (on 14% moisture basis) of the semolina were suspended in 100 mL of distilled water and heated in the visco-amylograph from 30 to 92 °C at a rate of 5 °C/min, held at 92 °C for 5 min, cooled to 50 °C at a rate of 5 °C/min and then held at 50 °C for 1 min under constant stirring (250 rpm). The torque measuring range was 300 cmg. The viscosity was expressed in Brabender units (BU). All analyses were performed in triplicate.

### 3.5. Phenolics

#### 3.5.1. Colourimetric Assay

Phenolic compounds were extracted according to Suriano et al. [35], with minor modifications. The samples (0.5 g) were extracted using 10 mL methanol (80:20) acidified with 1% 12 N HCl, for 30 min in an ultrasonic bath. After centrifugation, the supernatants were used for the determination of the total phenolic content (TPC), total flavonoid content (TFC), and antioxidant activity by ABTS assay.

Total polyphenol content (TPC) was determined using Folin–Ciocalteu reagent, according to the modified method of Suriano et al. [35] and expressed as µg gallic acid equivalents (GAE) g^–1^ DM. Total flavonoid content (TFC) was determined according to the method of Kim et al. [36] and expressed as µg catechin equivalents (CE) g^–1^ DM.

Total antioxidant activity (TEAC) was determined according to the method of Fares et al. [15], using a Trolox standard curve based on the percentage inhibition of absorbance at 734 nm and expressed as µmol Trolox equivalents (TE). All used reagents were obtained from Merk Life Science S.r.l, Milano, Italy. All chemical analyses were performed in triplicate.

#### 3.5.2. Extraction and Determination of Phenolic Acids

Soluble free and conjugated phenolic acids and insoluble bound phenolic acids were extracted, separated, and quantified according to the method described in Suriano et al. [35] with some modifications, by using an Agilent 1200 Series HPLC system (Agilent Technologies, Waldbronn, Germany) equipped with a diode array detector. The separation of phenolic acids was achieved using a reversed phase C18 column (InfinityLAB Poroshell 120 RC-C18, 100 × 2.1 mm; particle size = 2.7 μm) from Agilent (Santa Clara, CA, USA). The column temperature was at 35 °C, and the mobile phase consisted of (A) water with phosphoric acid 10^–3^ M and (B) acetonitrile at a flow rate of 0.5 mL/min, using the following linear gradient program: 5% B for 2.0 min, from 5% to 30% B for 10 min, from 30% B to 55% B for 1.0 min, from 55% to 70% for 2 min, isocratic at 70% for 1.0 min, linear gradient from 70% to 5% B for 6 min. Two microliters of sample were injected using an autosampler. The wavelengths used for quantification of the phenolic acids were 280 and 320 nm. The quantification was based on the peak area of the following standards: Gallic acid, *p*-Hydroxybenzoic acid, Vanillic acid, Caffeic acid, Syringic acid, Vanillin, Ferulic acid, Sinapic acid, *p*-Coumaric acid, Trans-cinnamic acid and Vitexin. Total phenolic acids (TPAs) were calculated as the sum of individual phenolic acids and expressed as µg g^−1^ DM. All chemical analyses were performed in triplicate. All used reagents were obtained from Merk Life Science S.r.l, Milano, Italy.

### 3.6. Statistical Analysis

A one-way analysis of variance (ANOVA) was applied to the dataset using the Statistica package (version 7.1, 2005; StatSoft Italia Srl, Vigonza, Italy). When significant differences (*p* < 0.05) were detected, Tukey’s honest significant difference (HSD) was computed. Pearson correlations (r) of the means among phenolics, morphological and qualitative traits were calculated. Principal component analysis (PCA) was applied to identify interrelationships between the varieties and the analysed parameters, i.e., morphological and yield-related characters, qualitative and bioactive compounds by a projection in a bi-dimensional scatter plot.

## 4. Conclusions and Future Perspectives

Although Cappelli is a low-yielding variety with poor gluten quality, it contains substantial levels of phenolic compounds and dietary fibre, particularly soluble dietary fibre, with prebiotic potential. Additionally, Marco Aurelio, which is a modern variety with intermediate yield potential, was found to have interesting properties such as the highest content in carotenoids and, among pasting properties, in peak viscosity. Moreover, it showed good levels of phenolic compounds in semolina. The other modern varieties are more oriented toward yield-related traits than phenolic compounds.

Interestingly, concerning the antioxidant characteristics, some individual phenolic acids were negatively correlated with certain yield-related traits such as grain morphology, thousand kernel weight and test weight.

Therefore, according to this study’s results, genotypes with low yield potential could accumulate higher phenolic acids with respect to high-yielding varieties under the same growing conditions, significantly contributing to durum wheat resilience of beneficial compounds. Especially, wholemeal was confirmed to be an effective way to introduce good quantities of bioactive compounds into the diet, with significant differences among varieties. Assessing the peculiar characteristics of such varieties, and promoting their consumption could be a good approach to increasing the daily intake of beneficial compounds, particularly dietary fibre and polyphenols.

Furthermore, we conclude the importance of carrying out further studies aimed at highlighting the effects of durum wheat selection programs based on nutraceutical characteristics, and suggest comparing genotypes through multi-local and year-replicate trials.

## Figures and Tables

**Table 1:** Image analysis of morphological parameters and yield-related characters in wholegrain of durum wheat genotypes.

Genotype	Length (mm)	Width (mm)	Thickness (mm)	TW (kg hL^−1^)	TKW (g)
Cappelli	7.33 ± 0.43 b	3.26 ± 0.36 c	2.99 ± 0.25 b	83.52 ± 0.1 c	47.71 ± 0.2 c
Sfinge	7.21 ± 0.58 b	3.49 ± 0.35 b	3.20 ± 0.25 a	85.84 ± 0.3 b	57.25 ± 0.3 b
Marco Aurelio	6.94 ± 0.44 c	3.09 ± 0.4 e	2.86 ± 0.26 c	80.05 ± 0.2 d	38.68 ± 0.1 d
Nadif	7.59 ± 0.52 a	3.61 ± 0.32 a	3.17 ± 0.25 a	87.36 ± 0.2 a	63.27 ± 0.2 a
Kronos	6.74 ± 0.56 c	3.17 ± 0.39 d	3 ± 0.29 b	82.95 ± 0.3 c	46.54 ± 0.4 c

Data are represented as the mean ± standard deviation of three replicates. The same letter indicates no statistical difference, whereas different letters stand for significant statistical difference (*p* < 0.05; Tukey’s test). Legend: TW = test weight; TKW = thousand kernel weight.

**Table 2:** Quality and phytochemical parameters in wholemeal of durum wheat genotypes.

Genotype	PC (g kg^−1^)	SDS (mL g^−1^)	YP (µg g^−1^ DM)	IDF (g 100 g^−1^)	SDF (g 100 g^−1^)
Cappelli	183 ± 0.1 a	23.9 ± 0.1 e	5.51 ± 0.05 e	10.86 ± 0.3 ab	3.34 ± 0.1 a
Sfinge	127 ± 0.2 c	31.2 ± 0.3 d	6.08 ± 0.01 d	11.22 ± 0.1 a	2.35 ± 0.1 bc
Marco Aurelio	179 ± 0.1 a	40.3 ± 0.3 a	11.28 ± 0.19 a	9.88 ± 0.1 c	2.55 ± 0.1 bc
Nadif	124 ± 0.2 c	35.2 ± 0.2 c	7.16 ± 0.10 c	10.21 ± 0.1 bc	2.14 ± 0.2 c
Kronos	160 ± 0.1 b	38.2 ± 0.3 b	8.71 ± 0.10 b	9.90 ± 0.3 c	2.90 ± 0.3 ab

Data are represented as the mean ± standard deviation of three replicate extractions. The same letter indicates no statistical difference, whereas different letters stand for significant statistical difference (*p* < 0.05; Tukey’s test). Legend: PC = protein content; SDS = SDS-sedimentation volume; YP = total carotenoid content; IDF = insoluble dietary fibre; SDF = soluble dietary fibre.

**Table 3:** Quality and phytochemical parameters in semolina of durum wheat genotypes.

Genotype	GC (g kg^−1^)	GI	YP (µg g^−1^ DM)	YI	RI	BI	Peak Viscosity (BU)	Breakdown (BU)	Setback (BU)
Cappelli	163.6 ± 0.05 ^a^	13.77 ± 0.24 ^d^	3.92 ± 0.01 ^e^	18.83 ± 0.11 ^d^	−0.95 ± 0.06 ^a^	14.60 ± 0.60 ^a^	874 ± 2.83 ^c^	86 ± 2.83 ^a^	583 ± 1.41 ^c^
Sfinge	107.3 ± 0.28 ^b^	50.20 ± 0.79 ^c^	4.80 ± 0.02 ^d^	19.58 ± 0.30 ^d^	−1.92 ± 0.08 ^b^	13.28 ± 0.67 ^a^	793 ± 1.41 ^d^	33 ± 1.41 ^c^	621 ± 2.83 ^b^
Marco Aurelio	154.6 ± 0.24 ^a^	88.98 ± 0.87 ^a^	9.32 ± 0.28 ^a^	28.40 ± 0.46 ^a^	−2.50 ± 0.06 ^c^	14.91 ± 0.52 ^a^	1054 ± 0.71 ^a^	5 ± 0.35 ^d^	370 ± 2.83 ^e^
Nadif	90.3 ± 0.81 ^c^	75.83 ± 0.82 ^b^	6.55 ± 0.02 ^c^	22.64 ± 0.19 ^c^	−3.16 ± 0.11 ^d^	13.70 ± 0.37 ^a^	1018 ± 1.41 ^b^	6 ± 0.71 ^d^	542 ± 2.83 ^d^
Kronos	148.6 ± 0.19 ^a^	87.18 ± 0.32 ^a^	8.44 ± 0.01 ^b^	26.21 ± 0.10 ^b^	−2.43 ± 0.13 ^c^	14.70 ± 0.06 ^a^	787 ± 2.83 ^d^	76 ± 2.83 ^b^	632 ± 1.41 ^a^

Data are represented as the mean ± standard deviation of three replicate extractions. The same letter indicates no statistical difference, whereas different letters stand for significant statistical difference (*p* < 0.05; Tukey’s test). Legend: GC = gluten content; GI = gluten index; total carotenoid content = YP; YI = yellow index; RI = red index; BI = brown index.

**Table 4:** List of the investigated genotypes.

Groups	Genotype	Taxonomic Classification	Year of Release	Pedigree/Country of Origin	Yield Potential
Old	Cappelli	*T. turgidum* ssp. *durum*	1915	Selection from the Tunisian population ‘Jean Retifah’-Italy	Low
Modern	Sfinge	*T. turgidum* ssp. *durum*	2003	Ofanto/Tavoliere//Doro–Italy	High
Modern	Marco Aurelio	*T. turgidum* ssp. *durum*	2010	Orobel//Arcobaleno/Svevo–Italy	Intermediate
Modern	Nadif	*T. turgidum* ssp. *durum*	2016	Claudio/Orobel–Italy	High
Modern	Kronos	*T. turgidum* ssp. *durum*	1992	APB MSFRS Pop selection D03–21–USA	Very high

## Data Availability

Not applicable.

## Supplementary Materials

The following supporting information can be downloaded at: https://www.mdpi.com/article/10.3390/plants12061350/s1, Table S1: Free Soluble phenolic, Soluble Conjugated, Insoluble Bound acids in wholemeal; Table S2: Free Soluble phenolic, Soluble Conjugated, Insoluble Bound acids in semolina.
