# Supplementary material for: Comparative Analysis of Qualitative and Bioactive Compounds of Whole and Refined Flours in Durum Wheat Grains with Different Year of Release and Yield Potential
Source: Plants (Basel). 2023 Mar 17;12(6):1350. doi: 10.3390/plants12061350 (PMC10053801; doi:10.3390/plants12061350)
Supplement: Supplementary file 1 [file plants-12-01350-s001.zip › plants-2229551-supplementary.pdf]

Supplementary materials.

**Table S1.**
Free Soluble phenolic, Soluble Conjugated, Insoluble Bound acids in wholemeal.

a) Free Soluble phenolic acids (μg $g^{-1}$) in wholemeal

| Genotype | Gallic acid | *p*-Hydroxybenzoic Acid | Vanillic acid | Caffeic acid | Syringic acid | Vanillin | Ferulic acid | Sinapic acid | *p*-coumaric acid | Trans-cinnamic acid | TPAs | Vitexin |
|---|---|---|---|---|---|---|---|---|---|---|---|---|
| Cappelli | 4.55$^{a}$ | n.d. | n.d. | n.d. | n.d. | 3.12$^{d}$ | n.d. | n.d. | n.d. | n.d. | 7.67$^{b}$ | 17.52$^{e}$ |
| Sfinge | 4.32$^{c}$ | n.d. | n.d. | n.d. | n.d. | 1.44$^{e}$ | n.d. | n.d. | n.d. | n.d. | 5.76$^{e}$ | 73.36$^{b}$ |
| Marco Aurelio | 4.08$^{b}$ | n.d. | n.d. | n.d. | n.d. | 3.36$^{c}$ | n.d. | n.d. | n.d. | n.d. | 7.44$^{c}$ | 75.04$^{a}$ |
| Nadif | 4.4$^{b}$ | n.d. | n.d. | n.d. | n.d. | 4.0$^{b}$ | n.d. | n.d. | n.d. | n.d. | 8.40$^{a}$ | 65.2$^{c}$ |
| Kronos | 2.72$^{d}$ | n.d. | n.d. | n.d. | n.d. | 4.32$^{a}$ | n.d. | n.d. | n.d. | n.d. | 7.04$^{d}$ | 54.88$^{d}$ |

b) Soluble Conjugated phenolic acids (μg $g^{-1}$) in wholemeal

| Genotype | Gallic acid | *p*-Hydroxybenzoic Acid | Vanillic acid | Caffeic acid | Syringic acid | Vanillin | Ferulic acid | Sinapic acid | *p*-coumaric acid | Trans-cinnamic acid | TPAs | Vitexin |
|---|---|---|---|---|---|---|---|---|---|---|---|---|
| Cappelli | n.d. | 1.455$^{d}$ | 6.95$^{d}$ | n.d. | 1.45$^{d}$ | 0.70$^{d}$ | 6.345$^{e}$ | 2.55$^{e}$ | n.d. | 4.25$^{c}$ | 23.71$^{e}$ | n.d. |
| Sfinge | n.d. | 2.345$^{a}$ | 8.75$^{a}$ | n.d. | 2.30$^{b}$ | 1.10$^{a}$ | 12.40a | 6.15$^{c}$ | n.d. | 3.60$^{d}$ | 36.65$^{a}$ | n.d. |
| Marco Aurelio | n.d. | 2.25$^{b}$ | 8.05$^{b}$ | n.d. | 2.40$^{a}$ | 0.90$^{b}$ | 10.10$^{b}$ | 10.245$^{a}$ | n.d. | 2.02$^{e}$ | 35.97$^{b}$ | n.d. |
| Nadif | n.d. | 2.045$^{c}$ | 7.50$^{c}$ | n.d. | 1.65$^{c}$ | 0.75$^{d}$ | 6.80d | 2.90$^{d}$ | n.d. | 5.165$^{b}$ | 26.81$^{d}$ | n.d. |
| Kronos | n.d. | 2.25$^{b}$ | 7.00$^{d}$ | n.d. | 2.45$^{a}$ | 0.80$^{c}$ | 7.145$^{c}$ | 7.10$^{b}$ | n.d. | 5.85$^{a}$ | 32.60$^{c}$ | n.d. |

c) Insoluble Bound phenolic acids (µg $g^{-1}$) in wholemeal

| Genotype | Gallic acid | *p*-Hydroxybenzoic Acid | Vanillic acid | Caffeic acid | Syringic acid | Vanillin | Ferulic acid | Sinapic acid | *p*-coumaric acid | Trans-cinnamic acid | TPAs | Vitexin |
|---|---|---|---|---|---|---|---|---|---|---|---|---|
| Cappelli | n.d. | 1.95$^{d}$ | 7.92$^{a}$ | 0.80$^{d}$ | 2.48$^{b}$ | 3.01$^{b}$ | 480.365$^{a}$ | 12.045$^{b}$ | 15.085$^{c}$ | n.d. | 523.65$^{a}$ | n.d. |
| Sfinge | n.d. | 2.10$^{c}$ | 6.10$^{c}$ | 1.18$^{b}$ | 2.63$^{a}$ | 2.93$^{b}$ | 450.58$^{c}$ | 12.93$^{b}$ | 17.20$^{a}$ | n.d. | 495.65$^{c}$ | n.d. |
| Marco Aurelio | n.d. | 2.68$^{b}$ | 7.18$^{b}$ | 1.04$^{c}$ | 2.13$^{d}$ | 3.22$^{a}$ | 469.46$^{b}$ | 10.12$^{c}$ | 15.29$^{b}$ | n.d. | 511.12$^{b}$ | n.d. |
| Nadif | n.d. | 2.90$^{a}$ | 5.53$^{e}$ | 0.57$^{e}$ | 2.24$^{c}$ | 2.51$^{d}$ | 421.23$^{d}$ | 7.11$^{d}$ | 11.31$^{d}$ | n.d. | 453.40$^{d}$ | n.d. |
| Kronos | n.d. | 1.55$^{e}$ | 5.78$^{d}$ | 1.37$^{a}$ | 2.22$^{cd}$ | 2.64$^{c}$ | 369.87$^{e}$ | 7.18$^{d}$ | 9.23$^{e}$ | n.d. | 399.84$^{e}$ | n.d. |

Results are expressed as µg $g^{-1}$ of flour on a dry sample weight basis. Data are represented as mean ±standard deviation of three replicate extractions. The same letter indicates no statistical difference, whereas different letters stand for significant statistical difference ($P < 0.05$; Tukey's test).

**Table S2.**

Free Soluble phenolic, Soluble Conjugated, Insoluble Bound acids in semolina

a) Free Soluble phenolic acids (μg $g^{-1}$) in semolina

| Genotype | Gallic acid | *p*-Hydroxybenzoic Acid | Vanillic acid | Caffeic acid | Syringic acid | Vanillin | Ferulic acid | Sinapic acid | *p*-coumaric acid | Trans-cinnamic acid | TPAs | Vitexin |
|---|---|---|---|---|---|---|---|---|---|---|---|---|
| Cappelli | 1.36[b] | n.d. | 1.52[a] | n.d. | n.d. | n.d. | n.d. | n.d. | n.d. | n.d. | 2.88[b] | 12.08[a] |
| Sfinge | 0.8[d] | n.d. | 1.12[c] | n.d. | n.d. | n.d. | n.d. | n.d. | n.d. | n.d. | 1.92[b] | n.d. |
| Marco Aurelio | 3.2[a] | n.d. | 1.36[b] | n.d. | n.d. | n.d. | n.d. | n.d. | n.d. | n.d. | 4.56[a] | 8[c] |
| Nadif | 0.88[e] | n.d. | 1.36[b] | n.d. | n.d. | n.d. | n.d. | n.d. | n.d. | n.d. | 2.24[c] | n.d. |
| Kronos | 1.205[c] | n.d. | 1.04[c] | n.d. | n.d. | n.d. | n.d. | n.d. | n.d. | n.d. | 2.25[c] | 10.4[b] |

b) Soluble Conjugated phenolic acids (μg $g^{-1}$) in semolina

| Genotype | Gallic acid | *p*-Hydroxybenzoic Acid | Vanillic acid | Caffeic acid | Syringic acid | Vanillin | Ferulic acid | Sinapic acid | *p*-coumaric acid | Trans-cinnamic acid | TPAs | Vitexin |
|---|---|---|---|---|---|---|---|---|---|---|---|---|
| Cappelli | n.d. | 0.555[c] | 1.325[c] | n.d. | n.d. | 0.405[c] | 1.365[e] | 2.38[c] | n.d. | n.d. | 6.03[c] | n.d. |
| Sfinge | n.d. | 1.01[b] | 2.03[b] | n.d. | n.d. | 0.09[e] | 2.285[c] | 2.99[b] | n.d. | n.d. | 8.41[b] | n.d. |
| Marco Aurelio | n.d. | 0.99[b] | 2.045[b] | n.d. | n.d. | 0.77[a] | 2.42[b] | 2.225[d] | n.d. | n.d. | 8.45[b] | n.d. |
| Nadif | n.d. | 0.445[d] | 1.25[c] | n.d. | n.d. | 0.32[d] | 2.045[d] | 2.4[c] | n.d. | n.d. | 6.46[c] | n.d. |
| Kronos | n.d. | 1.36[a] | 2.92[a] | n.d. | n.d. | 0.61[b] | 3.045[a] | 4.41[a] | n.d. | n.d. | 12.35[a] | n.d. |

c) Insoluble Bound phenolic acids (µg g$^{-1}$) in semolina

| Genotype | Gallic acid | *p*-Hydroxybenzoic Acid | Vanillic acid | Caffeic acid | Syringic acid | Vanillin | Ferulic acid | Sinapic acid | *p*-coumaric acid | Trans-cinnamic acid | TPAs | Vitexin |
|---|---|---|---|---|---|---|---|---|---|---|---|---|
| Cappelli | n.d. | 0.62[a] | 0.66[c] | n.d. | 0.32[b] | 1.87[b] | 63.7[a] | 1.14[c] | 1.675[c] | n.d. | 69.99[a] | n.d. |
| Sfinge | n.d. | 0.35[c] | 0.395[e] | n.d. | 0.24[d] | 1.91[ab] | 55.44[d] | 1.335[b] | 1.41[d] | n.d. | 61.08[c] | n.d. |
| Marco Aurelio | n.d. | 0.48[b] | 1.08[a] | n.d. | 0.725[a] | 0.43[d] | 59.845[b] | 1.45[a] | 2.045[a] | n.d. | 66.06[b] | n.d. |
| Nadif | n.d. | 0.23[d] | 0.59[d] | n.d. | 0.25[c] | 1.94[a] | 56.11[c] | 1.12[c] | 1.23[e] | n.d. | 61.47[c] | n.d. |
| Kronos | n.d. | 0.46[b] | 0.935[b] | n.d. | 0.3[bc] | 1.105[c] | 52.8[e] | 1.37[b] | 1.735[b] | n.d. | 58.71[d] | n.d. |

Results are expressed as µg g$^{-1}$ of flour on a dry sample weight basis. Data are represented as mean ±standard deviation of three replicate extractions. The same letter indicates no statistical difference, whereas different letters stand for significant statistical difference ($P < 0.05$; Tukey's test).
